# Is Mercury Absorbed by the Skin?

**Published:** 1881-05

**Authors:** 


### ARTICLE X.

### *Is Mercury Absorbed by the Skin?*

It is stated in Foster's Physiology that the balance of conflicting evidence is in favor of the view that soluble, non-volatile substances are not absorbed, and that volatile substances, as iodine, are not absorbed by the skin, but by the mucous membrane of the respiratory organs.

It is said that when salivation occurs from using mercurial ointments, it is because the particles of mercury are rubbed in the skin and thus reach the lymphatics. Prof. Furbinger has been making some experiments on this subject, which are given in full in *Virchow's Archin*, 82, iii, p. 491. The method employed, briefly stated, was to thoroughly rub the mercurial ointment on the uninjured skin, then wash the same and afterwards remove small pieces and harden them in alcohol. His experiments were conducted on various animals, including man. A careful microscopic examination of the skin was then made and particles of the mercury sought for. Globules were found all through the sebaceous glands and hair follicles, and some had entered the ducts of the sweat glands. He found that the vapor of mercury will not penetrate the skin at all, being deposited simply on the surface. In all cases no mercury globules were seen in the skin tissue itself; they were closely confined to the sebaceous glands and hair follicles. If, however, the skin was abraded and then an ointment of mercury applied, the globules would enter the tissue of the skin and also into the ruptured blood-vessels. The globules thus deposited in the glands of the skin lost their metallic lustre after a few days, becoming oxided, after which they were absorbed. Thus accounting for the persistent effects of mercurial innunctions.—*The Microscope.*

---

